# Bowel obstruction secondary to internal hernia in a hereditary angioedema patient: a case report

**DOI:** 10.1186/s12245-022-00475-9

**Published:** 2022-12-30

**Authors:** Atsuhito Tanaka, Ji Young Huh, Takamasa Yamamoto, Ken Washio, Koichi Ariyoshi

**Affiliations:** 1 Kobe City Medical Center General Hospital, 2-1-1 Minatojima-Minamimachi, Chuo-ku, Kobe, Hyogo Prefecture 650-0047 Japan; 2 Kobe City Nishi-Kobe Medical Center, 5-7-1 Kojidai, Nishi-ku, Kobe, Hyogo Prefecture 651-2273 Japan

**Keywords:** Hereditary angioedema, Internal hernia, Adhesions, Mechanical small bowel obstruction

## Abstract

**Background:**

Gastrointestinal attacks are frequent symptoms in patients diagnosed with hereditary angioedema (HAE). Cases of self-limited bowel intussusception and unneeded exploratory laparotomy due to lack of knowledge about HAE have been reported. Furthermore, after the introduction of C1-esterase inhibitor (C1-INH) concentrate, the recommended medication for HAE attacks, treatment has become typically medical in nature. We share a rare case where operative exploration was indicated to resolve a mechanical small bowel obstruction secondary to an HAE attack.

**Case report:**

An 80-year-old woman with HAE presented with lower left abdominal pain, vomiting, and nausea. Computed tomography (CT) showed edema of the small bowel and stomach as well as possible signs of mechanical small bowel obstruction. The patient was treated with C1-INH concentrate but showed only mild signs of relief, warranting diagnostic laparoscopy. Intraoperative findings showed internal herniation and strangulation of the small bowel caused by adhesions forming a band. After surgical intervention, no bowel resection was needed.

**Conclusion:**

Although C1-INH concentrate remains the principal treatment for HAE, gastrointestinal attacks may potentially cause surgical emergencies.

## Background

Hereditary angioedema (HAE) is a rare genetic disorder characterized by recurrent episodes of angioedema, which often affects mucosal tissue of the skin and respiratory and gastrointestinal tracts [1]. The most common gastrointestinal manifestations include nausea, vomiting, abdominal pain, and diarrhea. After the advent of C1-esterase inhibitor (C1-INH) concentrate, a treatment effective for HAE attacks, invasive procedures such as air-contrast enemas and abdominal surgery, which were previously performed due to underdiagnosis, are now avoided, making treatment typically medical in nature. We present a case where gastrointestinal symptoms from an HAE attack only showed mild relief after infusing C1-INH concentrate and warranted surgical intervention to remove the adhesion causing the internal hernia. We suspected that the adhesions were idiopathic, as the patient had no history of abdominal surgeries or any other obvious causes. Physicians need to be aware that although C1-INH concentrate remains a major treatment option, HAE may potentially cause bowel obstruction where surgical intervention is necessary.

## Case presentation

An 80-year-old woman with a history of HAE, angina pectoris, and cerebral infarction was referred to the emergency department due to severe pain in her lower left abdomen. She reported a one-day history of abdominal pain, nausea, and vomiting. She showed no other signs of edema of her skin or airway. Her previous attacks included abdominal pain and were occasionally accompanied by skin symptoms. Although she had experienced multiple HAE gastrointestinal attacks throughout her lifetime, they were all self-limited without administration of C1-INH concentrate. Her vital signs were a blood pressure of 163/65 mmHg, a heart rate of 68 beats per minute, and a temperature of 37.1 °C. On examination, the abdomen was soft and nondistended with tenderness and rebound in the left lower quadrant. She claimed that the pain was similar to her previous episodes of gastrointestinal attacks; however, her history of angina pectoris and cerebral infarction suggested increased vascular risks, making it necessary to exclude other diagnoses, such as mesenteric ischemia, mesenteric embolism, and ischemic enteritis.

**Fig. 1:**
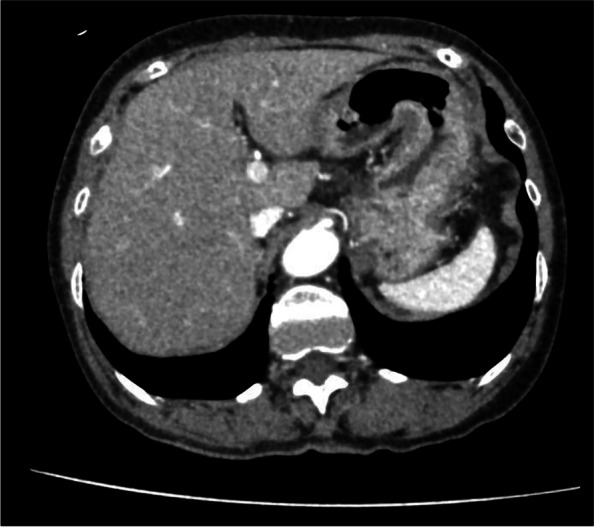
Edema of the gastric angle

**Fig. 2:**
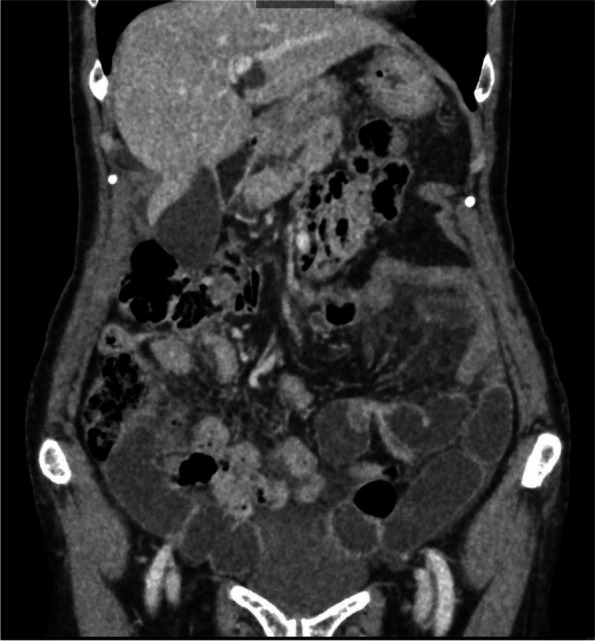
Internal hernia causing small bowel obstruction with mild ascites

Laboratory investigations showed an elevated white blood cell count of 17.4 × 10^5^/μL and a lactate concentration of 2.80 mmol/L. Other test results were unremarkable, including C-reactive protein, liver function, and electrolytes. Subsequent laboratory tests revealed a serum C4 level of 6 mg/dL (reference range: 20–50 mg/dL) and C1-INH activity as low as 15% (reference range: 70–130%). Computed tomography (CT) showed edema of the stomach, which was most pronounced at the gastric angle (Fig. 1), and mechanical obstruction of the proximal small bowel with mild ascites (Fig. 2). The latter indicated surgical intervention; however, the underlying cause was not evident. She had no abdominal distention, previous abdominal surgeries, obstipation or free air, suggesting that the HAE attack possibly caused the obstruction.

**Fig. 3:**
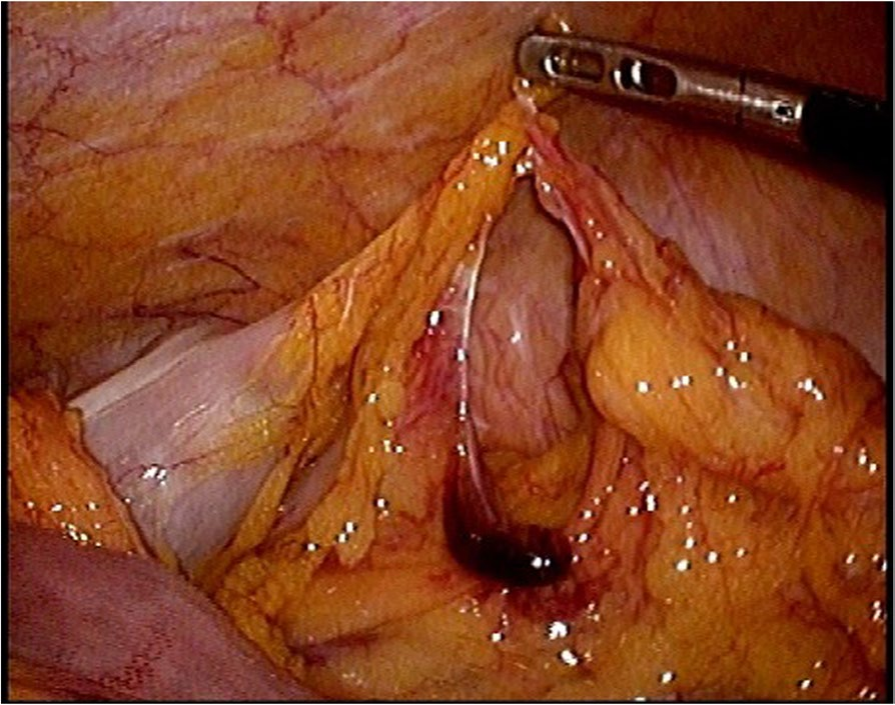
Intraoperative findings: transverse colon and sigmoid colon omental appendices forming a hernial orifice

Following guidelines suggesting C1-INH concentrate as the first-line choice for episodes of acute gastrointestinal HAE, the patient was administered an infusion of 1000 international units of C1-INH concentrate [2]. Typically, most patients show relief of abdominal and skin symptoms 15–20 min after treatment [3]. However, abdominal pain alleviation was mild even an hour after administration of the medication, while follow-up CT showed little change in the bowel obstruction, thus eliciting diagnostic laparoscopy. Intraoperative findings showed bloody ascites and a hernial orifice formed by the transverse colon and sigmoid colon omental appendices with the small bowel incarcerated (Fig. 3). Ligation of the hernial band showed instantaneous blood flow improvement in the bowel, eliminating the need for any bowel resection.

The patient was admitted to the intensive care unit (ICU) postoperatively due to possible laryngeal edema after extubation. However, she was successfully extubated; no further doses of C1-INH concentrate were necessary, and she was moved to the general ward. She was discharged with no apparent complications on hospital day 7. Subsequent laboratory tests showed elevation of C1-INH activity (45%) and serum C4 levels (16 mg/dL) compared with those observed on admission (15% and 6 mg/dL, respectively).

## Discussion

HAE is a rare autosomal dominant disorder, affecting 1 in 50–100,000 people of any ethnicity. The mean age at onset of symptoms is approximately 8–12 years, and swellings occur as frequently as once per week to less than once per year [4, 5]. HAE is categorized into two major types, with type I being the most common, accounting for 85% of cases, characterized by decreased concentrations of C1-inhibitor proteins. In 15% of HAE patients, normal or high concentrations of C1-INH, which is nonfunctional, are observed; this presentation is classified as type II [6]. Usually, C1-INH acts as an enzyme that downregulates the kallikrein-kinin system, adjusting the production of bradykinin. In HAE, where C1-INH is nonfunctional, increased levels of this inflammatory mediator are produced. This results in vasodilation, which presents as the typical symptoms of HAE [7]. HAE is clinically characterized by its lack of development of urticaria or pruritis and its effect on subcutaneous and submucosal tissues, leading to swelling of the upper respiratory tract and gastrointestinal system.

Gastrointestinal tract involvement is one of the most common features of HAE. Symptoms are present in 33–50% of patients, such as nausea, vomiting, abdominal pain, and diarrhea, which are the result of intestinal edema [1]. Symptoms can be nonspecific and may overlap with other abdominal conditions. In a review of 153 patients and 33,671 episodes of gastrointestinal attacks, less frequent symptoms were reported, including hemorrhagic diarrhea, tetany, and a single documented case of bowel intussusception [8]. There have been similar reports of small bowel obstruction and intussusceptions that resolved after infusion of C1-INH concentrate, but few have reported a bowel with impending ischemia [9, 10]. In this case, the surgical procedure revealed strangulation of the distal jejunum to ileum incarcerated as an internal hernia due to adhesions forming a hernial band. This suggests that a patient with adhesions can become symptomatic during a swelling attack, and the adhesions may need to be addressed surgically.

Abdominal adhesions are fibrous bands that span intra-abdominal organs. Although the mechanism of how adhesions are formed is unclear, 90% of adhesions form post-surgically. Other etiologies include radiation, formation secondary to inflammation such as endometriosis, pelvic inflammatory disease, Crohn’s disease and idiopathic formation [11, 12]. This patient had no prior abdominal surgeries, structural anomalies, or other medical backgrounds of abdominal inflammation other than multiple episodes of HAE gastrointestinal attacks, suggesting an idiopathic origin. Previous studies have shown that gastrointestinal attacks can be managed medically, and current treatment for acute attacks includes replacement of plasma-derived or recombinant concentrated C1-INH products, kallikrein inhibitor, and icatibant, a bradykinin B2 receptor antagonist [2]. Furthermore, hereditary angioedema of the gastrointestinal wall mimics a surgical emergency, and 30% of patients with HAE undergo inappropriate appendectomy, exploratory surgery, or both during abdominal attacks [6]. However, physicians need to be aware that HAE attacks can lead to surgical emergencies such as mechanical bowel obstruction and should not be completely dependent on pharmacological intervention to treat abdominal pain.

## Conclusion

HAE attacks often mimic other abdominal conditions, and unneeded procedures can be avoided by administering appropriate medication. Nevertheless, our report suggests that recurrent episodes of HAE may lead to a patient with adhesions becoming symptomatic due to internal hernia with an edematous bowel wall. Physicians need to be aware that abdominal pain from HAE attacks that does not resolve with C1-INH concentrate may have an underlying condition, including surgical emergencies.

## Data Availability

Data sharing is not applicable to this article, as no datasets were generated or analyzed during the current study.

## Abbreviations

HAE: Hereditary angioedema
CT: Computed tomography
C1-INH: C1-esterase inhibitor concentrate
ICU: Intensive care unit
